# Unmasking Rheumatic Heart Disease Decades Later in a Patient With a Remote History of Rheumatic Fever Despite Prolonged Antibiotic Prophylaxis

**DOI:** 10.7759/cureus.69367

**Published:** 2024-09-13

**Authors:** Samira Hayee, Maisha Maliha, Samsul Chowdhury, Nadera Naquib Bismee, Barna Dam, Sadia Afrin Naurin, Sananda Halder

**Affiliations:** 1 Internal Medicine, Albert Einstein College of Medicine, Jacobi Medical Center, New York City, USA; 2 Internal Medicine, Dhaka Medical College, Dhaka, BGD; 3 Internal Medicine, Queens Hospital Center, New York City, USA; 4 Internal Medicine, Sylhet MAG Osmani Medical College, Sylhet, BGD; 5 Internal Medicine, Uttara Adhunik Medical College Hospital, Dhaka, BGD; 6 Internal Medicne, Kumudini Women’s Medical College, Tangail, BGD; 7 Internal Medicine, Ibrahim Medical College, Dhaka, BGD

**Keywords:** aortic regurgitation, aortic stenosis (as), mitral regurgitation, mitral regurgitation (mr), mitral valve stenosis, rheumatic heart disease (rhd), rheumatic valvular heart disease, transcatheter aortic valve replacement (tavr), transesophageal echocardiography (tee), transthoracic echocardiogram

## Abstract

Rheumatic heart disease (RHD) is one of the leading causes of valvular heart disease worldwide and still persists in the USA, particularly among vulnerable populations with limited healthcare. Depending on the risk, severity, and types of valve involvement, treatment includes guideline-directed medical therapy (GDMT) and surgical interventions like valve repair or replacement. Here, we present a unique case of a patient in his late fifties who presented with worsening heart failure symptoms and several heart murmurs. A transthoracic echocardiogram (TTE) revealed moderate to severe mitral regurgitation (MR), aortic regurgitation (AR), and mild aortic stenosis (AS) with a bicuspid aortic valve. However, coronary angiography and right heart catheterization showed no blockages, right ventricular dysfunction, or pulmonary hypertension. Furthermore, no valvular vegetation was noticed on the transesophageal echocardiogram. The patient had a history of acute rheumatic fever (RF) in adolescence and was treated until age 21. Despite potential alternative causes like myocardial infarction or endocarditis, the lack of ischemic findings, negative blood cultures, and absence of valvular vegetation suggested that RHD was the possible cause of his valvular issues. This case highlights the rare occurrence of RHD impacting multiple valves despite proper antibiotic prophylaxis and draws attention to the importance of considering RHD when diagnosing multiple valvular problems, as many patients are identified too late for surgical intervention.

## Introduction

Rheumatic heart disease (RHD) is becoming rare in developed countries; however, it is still a significant problem worldwide. Rheumatic fever (RF) is an acute, immune-driven inflammatory disorder affecting multiple body systems, triggered by a prior infection with Group A streptococcus (GAS), most commonly following a throat infection. Furthermore, RHD is a long-term condition that arises from the inflammation caused by RF, primarily affecting the heart. The most notable feature of RHD is damage to the heart valves, which can lead to persistent issues such as heart failure, atrial fibrillation, and a higher risk of stroke. As the most severe outcome of RF, RHD can cause lasting harm to the heart valves [1].

Globally, RHD is still the most predominant cardiovascular disease, affecting individuals younger than 25 years old. While RF and RHD have been eliminated mainly in regions with developed economies, the movement of people from low-income to high-income areas could potentially lead to a resurgence of RHD cases in high-income nations [2]. From 2000 to 2012, the estimated incidence of RF in children across the United States was 0.61 cases per 100,000 children [3]. One recent study revealed that there has been an increase in RHD incidence across most high-income countries in Europe for both sexes, notably beginning around 2014. This rise aligned with the increased migration and refugee movement into Europe. The observed increase in RHD rates varied from +0.4% to +24.7% for males and +0.6% to +11.4% for females [4]. Usually, there is a transition period between rheumatic carditis in acute RF and RHD with chronic valvular disease. Rheumatic heart disease and permanent heart valve damage usually evolve over the years after single or recurrent attacks of acute rheumatic fever (ARF) [5]. However, a population-based study from Uganda described that most patients diagnosed with RHD lack a history of ARF. This can also be due to a missed diagnosis [6]. The Global Rheumatic Heart Disease Registry (the REMEDY study) revealed that, in the post-antibiotic era, isolated mitral regurgitation (MR) is the commonest among young patients <20 years, while mitral stenosis is more common after 20 years. On the other hand, the elderly population usually presents with multivalvular lesions [7]. Rheumatic heart disease can lead to cardiovascular complications, particularly acute decompensated heart failure, valvular abnormalities, conduction anomalies, and increased morbidity and mortality. The hemodynamics associated with the progression of RHD demand increased monitoring and better management. 

Management of RHD depends on the disease severity, risk stratification, and the type of valves involved. However, the significance of the modality of valvular surgery is a field that is not widely explored. The parameters that determine a patient's eligibility for a valvular replacement and the factors determining the success and mortality rates in the procedure are topics that need more research and trials. For example, according to the guidelines of the European Society of Cardiology (ESC), surgery is indicated in acute severe MR and symptomatic severe primary MR with acceptable surgical risk according to the decision of the Heart Team. On the other hand, if the aortic regurgitation (AR) is severe, surgery is recommended. The operative risk is not a contraindication for symptomatic AR [8].

Here, we describe a case of RHD with multiple ventricular involvements in the present day and age in a developed country and how different hemodynamic parameters determined his eligibility for surgery and modalities of treatment.

## Case presentation

We present a case of a patient in his late fifties who presented to the emergency department at NYC Health & Hospital due to worsening dyspnea on exertion over two to three months. The severity of the dyspnea reduced his functional exercise capacity to walk half a block from several blocks, and it worsened with exertion, such as walking or climbing a flight of stairs. In addition, the patient experienced orthopnea and paroxysmal nocturnal dyspnea as he could not tolerate lying flat, which forced him to use a recliner to sleep. The patient also complained of atypical left-sided chest pain, which was sharp with a severity of 5/10, worsening with exertion and relieved by rest.

The patient was originally from the United States. In his relevant medical history, he was diagnosed with RF in his teens after suffering multiple attacks of fever and sore throat, suggesting possible acute episodes of RF. The patient had been on antibiotic prophylaxis with amoxicillin until the age of 21 years, and he was adherent to his medication. He reported completion of treatment. However, the patient had not been in follow-up with a cardiologist after that. Additionally, the patient was a smoker and quit one year ago, accumulating seven pack years. Patient did not follow up with a cardiologist during the third and fourth decade of his life as he did not have any cardiac symptoms.

On examination, the patient showed telltale signs of volume overload and rheumatic valvular heart disease. There was bilateral 2+ pitting edema up to the knees, elevated jugular venous pressure (JVP), and crackles in both lung basal areas. On auscultation of the chest, there was a blowing systolic murmur, grade 3/6, in the apical region, heard best in the left lateral position, suggesting MR. There was a 3/6 decrescendo diastolic along the mid-right sternum, along with a 2/6 crescendo-decrescendo murmur systolic murmur in the suitable second intercostal space, radiating to the neck bilaterally with no parvus and mild tardus, pointing towards a mixed valvular disease of aortic stenosis (AS) and AR.

In summary, the 50-year-old male presented with worsening dyspnea on exertion, orthopnea, paroxysmal nocturnal dyspnea, and atypical chest pain, alongside physical signs of volume overload and multiple heart murmurs indicative of valvular disease.

Relevant investigations

**Figure 1:**
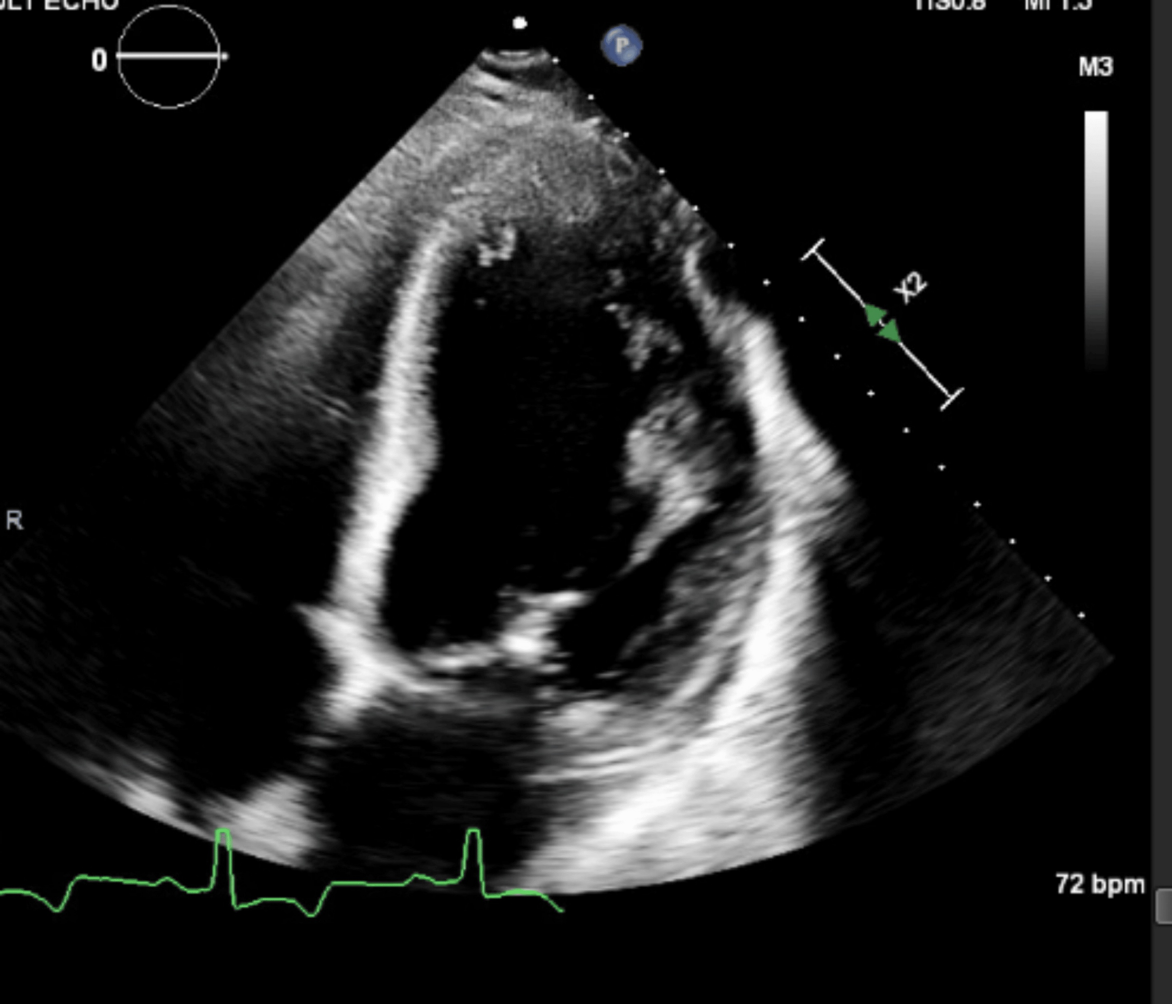
Transthoracic echocardiography images showing left atrial and ventricular dilatation and hypokinesis, indicating severe cardiac remodeling consistent with chronic rheumatic heart disease

Initially, a transthoracic echocardiogram (TTE) was done, which showed ejection fraction (EF) to be 27%, with severely diffuse left ventricular hypokinesis, grade II diastolic dysfunction (E/A 2), and severe dilation of the left atrium (LAESV index-54 ml/m2 and E/e'15), as can be seen in Figure 1. These findings were indicative of severe myocardial dysfunction, likely secondary to chronic RHD. The observed changes explain the patient's symptoms of heart failure.

**Figure 2:**
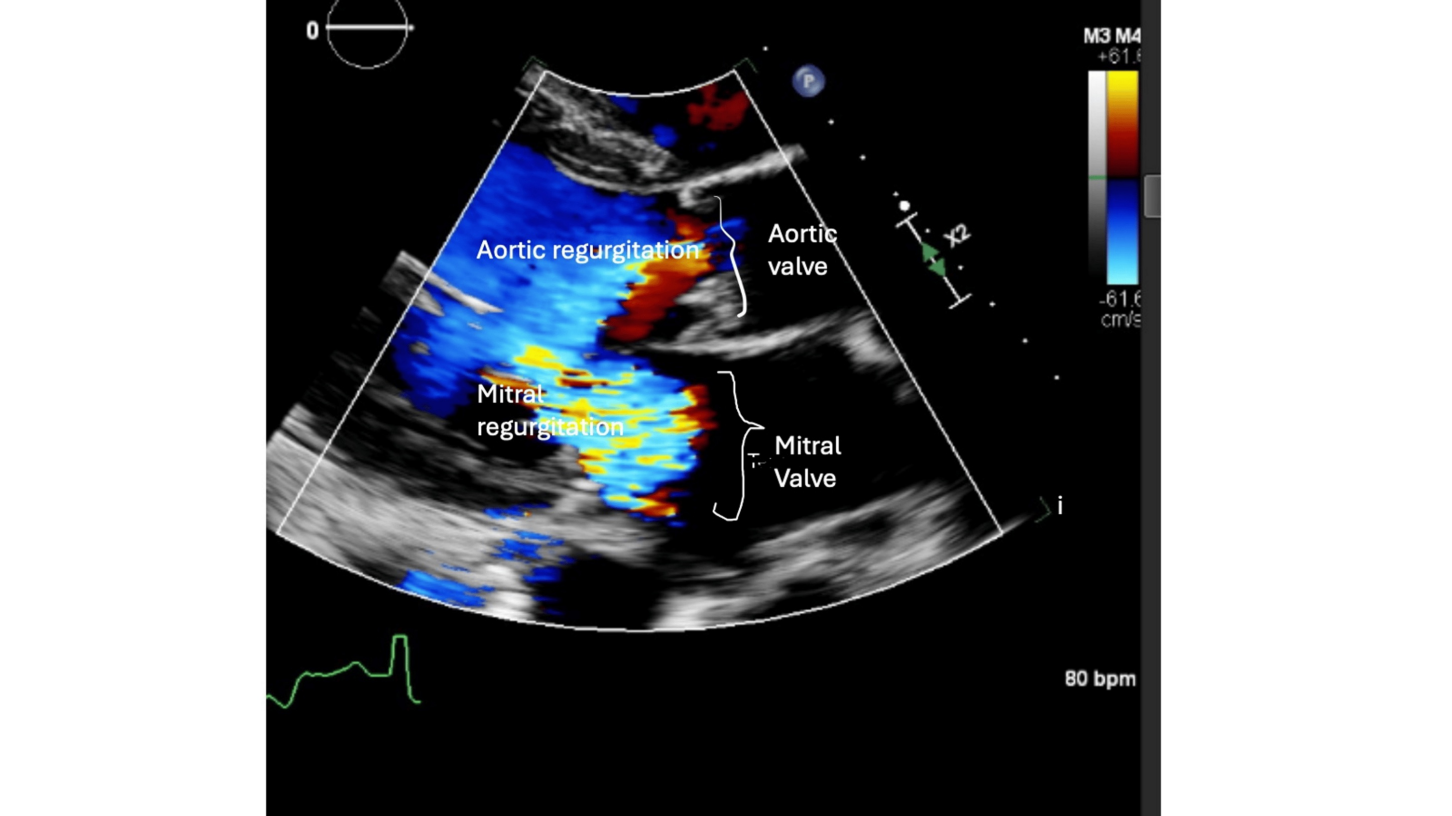
Transthoracic echocardiography image of aortic and mitral valve dysfunction and insufficiency (Doppler mode) These findings correlate with the patient's symptoms of dyspnea and orthopnea, indicating significant valvular disease.

**Figure 3:**
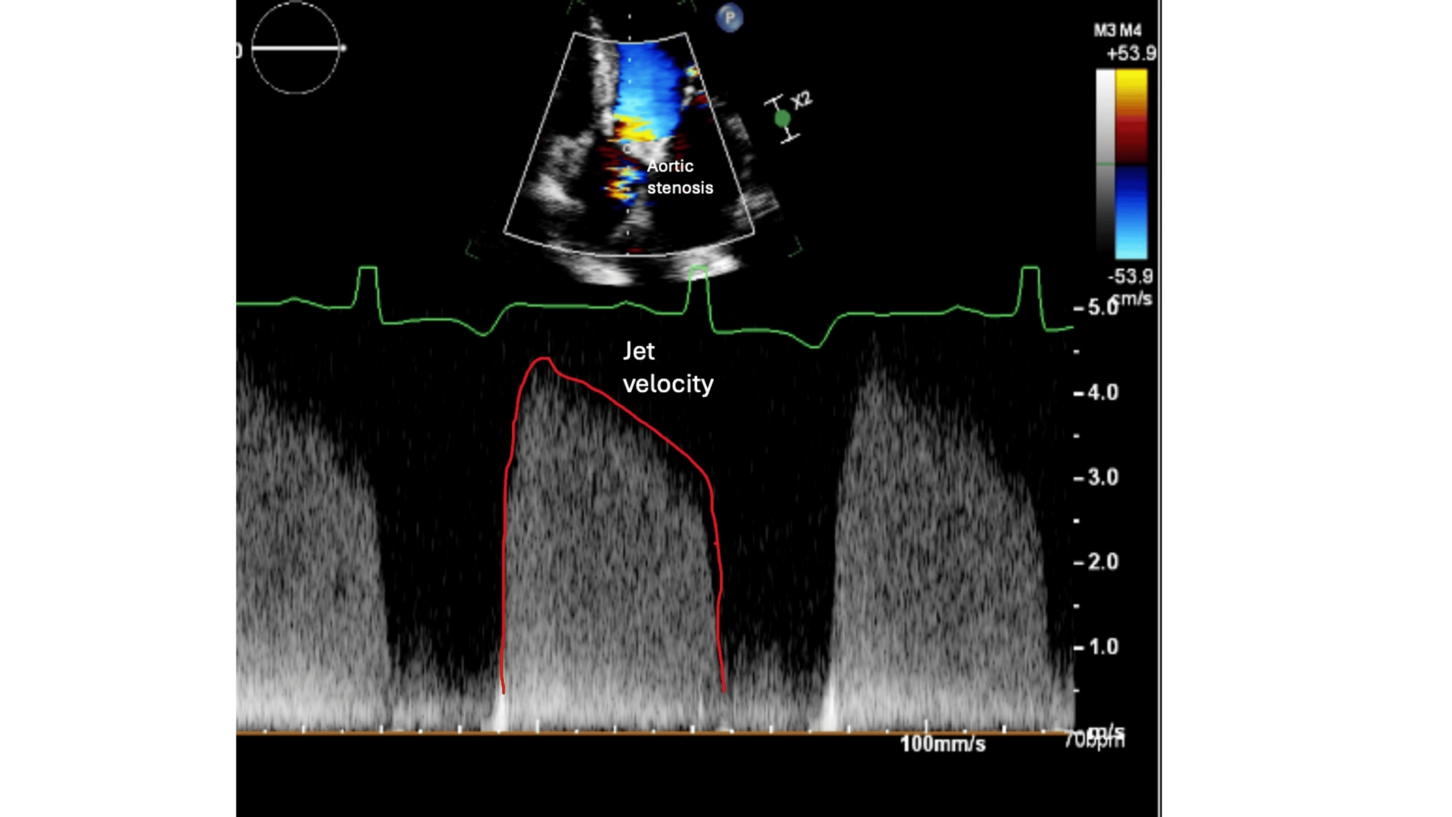
Transthoracic echocardiography image showing jet velocity across the aortic valve The increased velocity suggests significant aortic stenosis, which, in conjunction with the regurgitation observed, points to severe mixed valvular disease.

Furthermore, there was moderate to severe MR with annular dilation, moderate to severe AR, and moderate aortic valve stenosis with bicuspid aortic valve parameters, including aortic valve area (AVA) of 1.1 cm^2^ and mean trans-valvular gradient of 21 mmHg, as can be seen in Figures 2-3, suggesting significant valvular abnormalities.

There was suspicion of low flow, low gradient aortic stenosis in the setting of low EF in the TTE findings. Hence, dobutamine-induced stress echocardiography was done; 15 mcg of dobutamine was infused, which increased EF to 35%. In the presence of dobutamine, the parameters for mild AS changed to moderate AS, in which AVA increased to 1.3 cm^2^, and the mean transvalvular gradient increased to 30 mmHg. Hence, the diagnosis of moderate to severe multiple valvular dysfunction was confirmed. Furthermore, to properly visualize the valvular anatomy, a transesophageal echocardiogram was performed, which showed a small calcified mobile linear mass in the non-coronary sinus in the aortic area measuring 9 mm x 1 mm and an immobile calcified mass lower in the sinus, possibly affixed to the leaflet.

Coronary angiography and right heart catheterization showed no obstructive lesions, right ventricular dysfunction, or pulmonary hypertension.

**Figure 4:**
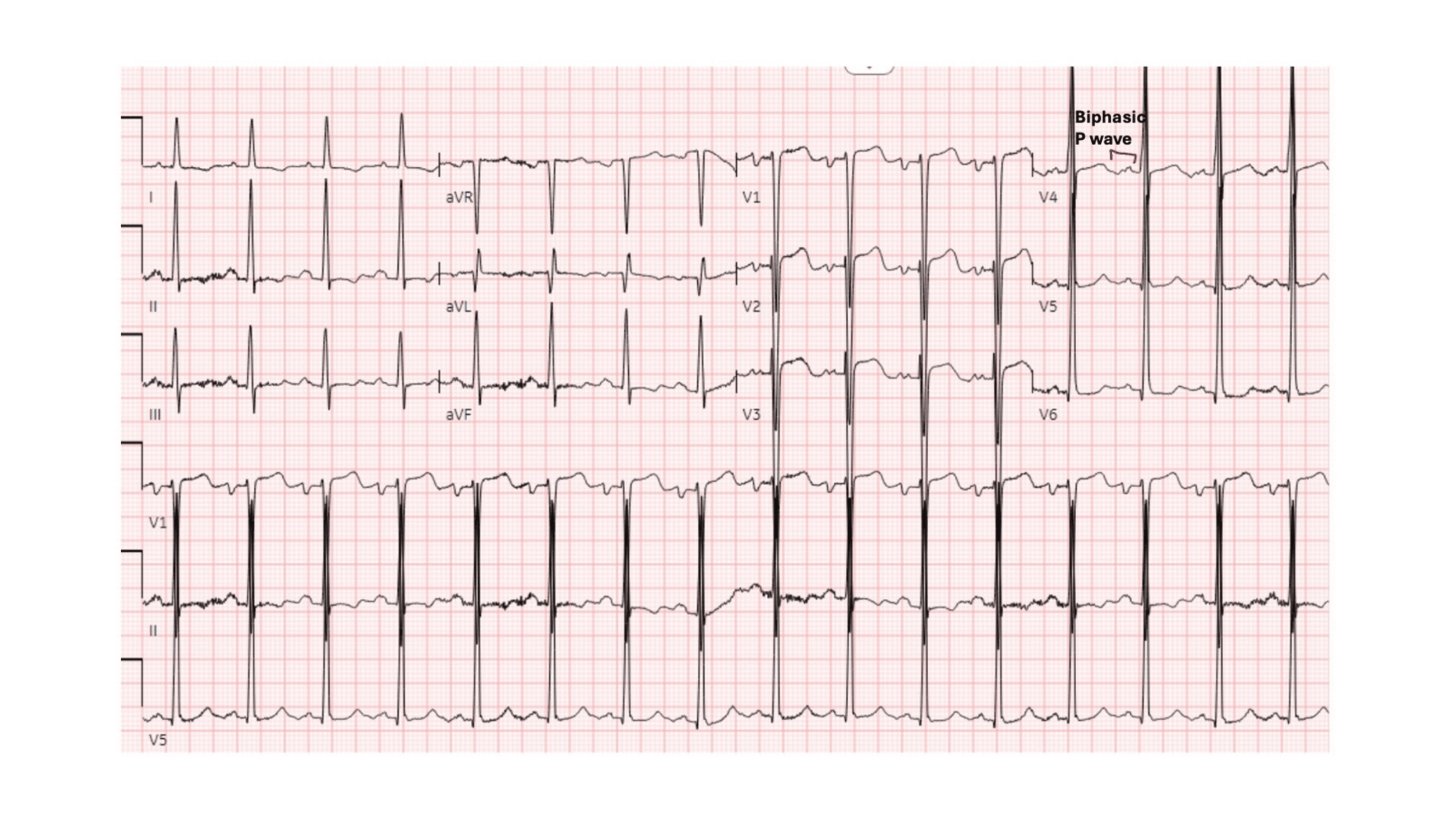
The EKG changes are suggestive of left ventricular hypertrophy and left atrial enlargement Left atrial enlargement is represented by P waves being bifid in the lead II and being 1 mm above and across in V1, and left ventricular hypertrophy according to Sokolov-Lyon criteria—the S wave in V1 and the R wave in V5 and V6 being >35 mm.

The chest x-ray showed signs of small fluid overload. An ECG showed signs of left atrial enlargement (P waves being bifid in the lead II and being 1 mm above and across in V1) and left ventricular hypertrophy (Sokolov-Lyon criteria-S wave in V1 and R wave in V5 and V6 being >35mm), as can be seen in Figure 4. Tests such as syphilis and blood culture to rule out infectious causes revealed nothing significant.

Differential diagnosis

Other possible etiologies for acute decompensated heart failure with reduced EF complicated by valvular pathology could be due to ischemia such as myocardial infarction, endocarditis, or valve prolapse. However, given the history of ARF in childhood and the lack of coronary obstruction in coronary angiography or signs of ischemic changes on EKG, negative blood cultures, and lack of vegetation seen on TEE, other possible diagnoses were highly unlikely.

Treatment

The treatment for acute decompensated heart failure with reduced EF was started. Furosemide 20 mg IV was begun, followed by initiation of guideline-directed medical therapy (GDMT). Valsartan-sacubitril 24-26 mg, spironolactone 25 mg, and empagliflozin 10 mg were started. Beta-blockers were held off until optimization of volume status. He met the criteria for surgical valvular replacement, which was deferred till the optimization of the GDMT outpatient. He was discharged with regular follow-ups with outpatient cardiology.

Outcome and follow-up

The patient showed marked improvement with the initiation and optimization of treatment. His dyspnea subsided with significantly improved exercise tolerance and quality of life. He is currently being followed by outpatient cardiology with optimization of GDMT for his heart failure. The plan is to optimize GDMT for his heart failure with non-ischemic cardiomyopathy with a repeat TTE in three months to see improvement in his ejection fraction and valvular pathology. If his moderate to severe valvular pathology persists even after improving his EF and left ventricle function, the plan for cardiothoracic surgery for valve replacement will be pursued. The patient is amenable to surgery and is adherent to his medications.

## Discussion

Rheumatic heart disease, sequelae of RF, is an immunological non-suppurative manifestation of group A beta-hemolytic streptococcal pharyngitis [9]. Recurrent attacks of RF lead to irreversible, profound damage to cardiac valves and result in RF [10]. Better access to medical care and penicillin prophylaxis have led to a marked decline in the presence of RHD [10]. A recent nationwide study in Sweden demonstrated that RF was rare in developed countries [11]. One exception to this norm is the patient in this study who, despite completing the antibiotic prophylaxis dosage till 21 years of age and being born and raised in the US, developed severe symptomatic RHD and acute decompensated heart failure with reduced EF [11].

The World Heart Federation (WHF) has created echocardiographic criteria for diagnosing RHD. These criteria classify individuals into three groups based on 2D continuous-wave and color Doppler echocardiography: 'definite RHD,' 'borderline RHD,' and ‘normal’. Patients with a confirmed history of ARF or any observed structural or functional valvular issues should be initially regarded as having RHD unless proven otherwise [11]. The WHF has defined subcategory C as pathological aortic regurgitation and at least two morphological features of RHD of the aortic valve. Subcategory D is defined as a borderline disease of both the aortic and mitral valves [12]. Our patient falls into Subcategory C (RHD of the AV) because of AR and morphological features of the valve in RHD, and Subcategory D (multivalvular RHD) because of illness of both the aortic and mitral valve.

Many patients later diagnosed with rheumatic valvular heart disease arrive at the hospital when surgical intervention is no longer a viable choice [13]. However, in our case, the patient was eligible for surgical interventions given the presence of moderate to severe valvular pathologies. According to the 2020 American Heart Association (AHA) guidelines, it is a Class 1 indication to perform aortic valve surgery for severe symptomatic aortic regurgitation; a Class 2b indication to undergo valve replacement for moderate AS who are undergoing cardiac procedures for other indications, with surgical aortic valve replacement (SAVR) being preferred over transcatheter aortic valve replacement (TAVR) for bicuspid aortic valve; and a Class 1 indication to perform intervention for severe MR with mitral valve repair preferred over mitral valve replacement [14]. According to ESC guidelines 2021, it is a Class I B indication to perform aortic valve surgery for symptomatic patients regardless of left ventricular function. Additionally, mitral valve repair surgery is a Class I Level B recommendation for symptomatic patients who are suitable candidates for surgery and not at high risk. Furthermore, surgery is advised for asymptomatic patients with left ventricular dysfunction, indicated by a left ventricular end-systolic diameter (LVESD) greater than 40 mm and/or a left ventricular EF less than 60% [15]. However, the decision of choosing over the various modalities of treatment, such as choosing SAVR or TAVR for AS, mitral valve repair or replacement for MR, depends on decisions decided by a multidisciplinary team of interventional cardiologists, structural heart disease specialists, imaging specialists, and the patient's profile and wishes. Nevertheless, the usual first step in managing symptomatic RHD is always medical management to effectively correct the left ventricle systolic dysfunction, which was done with fruition in this patient [16]. Interestingly, there have been newer interventional approaches for managing severe MR. For example, transcatheter edge-to-edge repair (TEER) is a relatively novel alternative to surgery for treating severe MR. Recent randomized trials have shown that mitral TEER is safe and effective in treating symptomatic patients with severe MR, particularly in functional and also in degenerative diseases. Based on the positive findings of these studies, the ESC guidelines on valvular heart disease recommend mitral TEER as an option for managing secondary MR with a class II recommendation in patients experiencing a significant decline in their functional capacity, despite receiving optimal, maximum tolerated guideline-directed pharmacotherapy [17].

Although the prevalence of RHD has decreased markedly over the last century, it still has a global majority of around 15.6 million cases worldwide [18]. A recent study showed that RHF is still a significant cause of morbidity and mortality in developed regions with the progression of RF to RHF, even after extended periods of prophylaxis [19]. Many new advances are being undertaken to reduce the burden of RHF. There have been advances in the development of vaccines against GAS with StreptAvax:26 valent vaccines showing much promise in trials, better diagnostic tools such as nucleic acid amplification tests with advances in machine learning and artificial intelligence with throat image processing, and advances in the management of valvular pathologies such as TAVR and balloon valvulotomy [16]. 

Learning points/take-home messages

Although the prevalence of RHD has been decreasing over the last decade, it is still present in developed countries and should be elicited from the patient's history in the background of valvular pathology and heart failure.

There are many modalities to the treatment of RHF, which include GDMT to manage heart failure, surgical interventions for valvular pathologies such as TAVR or SAVR for AS, mitral valve repair or replacement for MR, percutaneous balloon valvulotomy for MS, and aortic valve replacement for AR.

Research and advances are being undertaken for better primary prevention and better detection of RHD, such as vaccines against GAS and throat image tests.

The parameters that determine the eligibility of a patient to get a valvular replacement and the factors determining the success and mortality rates in the procedure are topics that need more research and trials, and patients with a history of RF should always remain in proper follow-up for the risk of developing RHF.

## Conclusions

Despite a decline in the prevalence of RHD over the past decade, it remains a concern in developed countries and should be considered as a differential in patients with valvular pathology and heart failure. Rheumatic heart disease should be kept in consideration when multi-valvular involvement is seen, especially in patients who migrated from other countries, although our patient did not move from another country or travel outside the US. Many patients are diagnosed with rheumatic valvular heart disease at a stage when surgical options are no longer viable. Further studies are needed to refine the criteria for valvular replacement eligibility and to better understand the factors influencing success and mortality rates in these procedures. Patients with a history of RF should maintain regular follow-up due to the risk of developing RHD.
